# College students’ life stress and internet addiction: the roles of loneliness and physical exercise

**DOI:** 10.3389/fpsyg.2026.1858975

**Published:** 2026-06-17

**Authors:** Guoqiang Song, Wenkai Wang, Shuang Zhao, Xingyu Yi, Wanlei Lin

**Affiliations:** 1 Physical Education Institute, Hanjiang Normal University, Shiyan, China; 2 Physical Education Department, Handan Preschool Teachers College, Handan, China; 3 Physical Education Institute, Hubei University of Arts and Science, Xiangyang, China

**Keywords:** college students, internet addiction, life stress, loneliness, physical exercise

## Abstract

**Objective:**

This study investigates the relationship between life stress (LS) and Internet addiction (IA) among college students, focusing on the mediating role of loneliness (LL) and the moderating role of physical exercise (PE). A moderated mediation model was developed based on stress-coping theory and self-regulation theory.

**Methods:**

A survey was conducted with 409 college students from various regions, and statistical analyses were performed using SPSS 28.0 and Process 4.1.

**Results:**

(1) Life stress, loneliness, and Internet addiction were significantly positively correlated, whereas physical exercise exhibited a significant negative correlation with all three variables; (2) Loneliness served as a partial mediator in the relationship between life stress and Internet addiction, with a mediating effect value of 0.248, representing 40.7% of the total effect; (3) Physical exercise negatively moderated the relationships between life stress and Internet addiction (*β* = −0.366, *p* < 0.01), life stress and loneliness (*β* = −0.348, *p* < 0.01), and loneliness and Internet addiction (*β* = −0.341, *p* < 0.01).

**Discussion:**

Life stress and loneliness are identified as risk factors for Internet addiction among college students, while physical exercise emerges as an effective intervention strategy.

## 1 Introduction

The rise of large language models and the boom of the short-video era have brought a lot of convenience and fun to life and work, but they have also given rise to a series of potential risks, such as the weakening of hands-on and cognitive abilities, the prominent problems of sedentary behavior and sub-health, which continuously drive up the incidence of Internet addiction. Especially for today’s college students, due to their relatively weak self-control ability and the convenience of Internet use, Internet addiction has become one of the important factors affecting their own development (Dong et al., 2024). The World Health Organization defines Internet addiction as a behavioral disorder in which an individual loses control over Internet use and suffers from listlessness due to excessive dependence (Bakioglu, 2020; Liu et al., 2025b). Relevant statistics show that the number of Internet users in China has reached 1.079 billion, with teenagers accounting for 17.7%, and the prevalence of Internet addiction among college students is close to 12% (Nibal, 2023), which seriously restricts their quality of life and academic performance (Xu and Tang, 2024). Another survey, “2026 China Youth Emotion Survey Report”, shows that 97% of young people perceive different levels of life stress, and 73.9% of young people have different levels of loneliness.

Today’s youth have fallen into a double situation of “anxiety + loneliness” (Zheng et al., 2026). Therefore, systematically exploring the influence mechanism of life stress on college students’ Internet addiction is both urgently needed in the current era and of practical importance.

Current research on college students’ Internet addiction mainly focuses on dimensions such as harm manifestations, inducing factors, and intervention strategies (Mousoulidou et al., 2024). Among the many inducing factors, negative emotions have been proven to be important contributing factors (Huang et al., 2025). For example, the study by (Shen et al., 2023) pointed out that there is a significant positive correlation between anxiety, life stress, and Internet addiction. This indicates that college students experiencing a high level of life stress are more likely to exhibit excessive or uncontrollable Internet use behaviors. According to the stress-coping theory, when individuals perceive a lack of psychological resources (such as interpersonal support), they tend to adopt avoidance coping strategies (Mousoulidou et al., 2024). Based on this, it can be inferred that loneliness may play a mediating role between life stress and Internet addiction. Loneliness is an experience of unmet social needs, stemming from dissatisfaction with one’s social relationships (Zhou et al., 2025), and can be regarded as a sign of psychological resource depletion. However, existing research has not fully explored the mediating effect of loneliness between life stress and Internet addiction. In addition, relevant studies have shown that regular physical exercise can not only significantly improve college students’ well - being and effectively relieve the level of psychological stress (Xu and Tang, 2024), but also directly intervene in Internet addiction behaviors (Xu and Tang, 2024), and reduce the level of loneliness by increasing peer communication and peer assistance (Zhou et al., 2025). However, the path of physical exercise in regulating the relationship among life stress → loneliness → Internet addiction has not been empirically verified. Based on the above research gaps, it has become an urgent need in current research to deeply explore the mediating and moderating roles of loneliness and physical exercise in the relationship between college students’ life stress and Internet addiction.

## 2 Literature review

### 2.1 The relationship among life stress, loneliness, and internet addiction

Life stress refers to the mental state of tension and anxiety experienced by individuals when they are confronted with threatening or challenging events in daily life or study, and their own resources (such as ability, time, experience, material, and social connections) are insufficient to cope with these events (Yan et al., 2022). In this study, life stress refers to the subjective feeling of pressure that individuals experience when dealing with various life situations. It is divided into two dimensions: sense of tension and sense of loss of control, that is, the degree to which individuals feel emotionally excited, difficult to relax, and unable to cope with pressure (Yan et al., 2022; Yang et al., 2022a). College students are a group that has just escaped from the strict control of their parents. Due to issues such as sensitivity in interpersonal relationships, increased employment pressure, incomplete economic independence, weak self-control, and strong herd mentality, they are a high-risk group prone to psychological problems (Ye et al., 2025). A study shows that stress, self-control, anxiety, and self-efficacy are all predictors of Internet addiction, among which stress plays a role through the indirect path of anxiety and self-control (Chen and Zhang, 2024). Based on the compensatory Internet use theory, researchers point out that when individuals face a high level of perceived stress, they tend to use the Internet as a means to escape reality and regulate negative emotions (Song et al., 2004). In addition, negative life events have also been proven to have a significant positive correlation with Internet addiction among college students. Pro-social behavior plays a mediating role in this process, and physical exercise plays a regulatory role in some paths (Huang et al., 2025).

Loneliness, as a predictor of internet addiction, has begun to attract the attention of scholars in recent years. It refers to the emotional experience of individuals subjectively feeling isolated and alienated from others and society, and the emotional state in which individuals are unable to express their emotions and feel inner emotional loss (Pierce et al., 2003; Maes et al., 2016). A cross-sectional study by Koktas et al. (2024) found that loneliness, self-esteem, and life satisfaction were predictors of internet addiction among Turkish college students. The higher the level of loneliness, the greater the risk of internet addiction. Li et al. (2024) used Chinese college students as a sample to verify the mediating role of loneliness between psychological resilience and internet addiction. They found a significant positive correlation between loneliness and internet addiction, and a negative correlation between psychological resilience, life satisfaction, and internet addiction. A meta-analysis by Wang and Zeng (2024a) integrated multiple studies and confirmed a robust positive correlation between loneliness and internet addiction. Moreover, existing studies have shown that loneliness has a significant mediating role between physical exercise and internet addiction (Lv et al., 2025a).

Although the existing literature has fully explored the pairwise relationships among life stress, loneliness, and Internet addiction, studies that take loneliness as a mediator between life stress and Internet addiction are rare, especially those targeting the college student population. Therefore, this study proposes Hypothesis 1: Loneliness mediates the effect of college students’ life stress on Internet addiction.

### 2.2 Moderating effect of physical exercise

Relevant studies have shown that physical exercise can not only improve the physical health of college students but also have significant effects in improving negative emotions and bedtime procrastination (Netz and Raviv, 2002). Multiple studies have revealed the buffering effect of physical exercise in the processes of negative emotions, Internet addiction, and bedtime procrastination from different perspectives. For example, a moderated mediation model analyzed the differential moderating effects of different levels of physical exercise on the relationship between negative life events and Internet addiction, revealing that physical exercise can weaken the inducing effect of negative life events on Internet addiction (Huang et al., 2025). Another study, through a survey of 2,892 Chinese college students, found that moderate-intensity and high-intensity physical activities play a partial mediating role between college students’ stress and Internet addiction, while low-intensity physical exercise has no significant effect (Luo et al., 2024). This non-linear relationship strengthens the understanding of the boundary effect of physical exercise, indicating that different intensities of physical exercise have different buffering effects.

The moderating effect of physical exercise not only acts on the direct path between life stress and Internet addiction but may also extend to the mediating path of loneliness. Relevant research indicates that physical exercise can influence Internet addiction through various psychological mechanisms. For example, in the study by Wang et al., by constructing a structural equation model, it was found that physical exercise not only has a negative impact on Internet addiction among college students but also affects Internet addiction through the mediating effect of loneliness, with the mediating effect accounting for 9.38%. Similar to this, the study by Xu and Tang (2024) confirmed that physical exercise can prevent Internet addiction behavior among college students through the chain mediating path of enhancing self-control and weakening loneliness. In conclusion, physical exercise can alleviate Internet addiction behavior by reducing the level of loneliness or suppressing life stress. On the one hand, it may alleviate Internet addiction by enhancing psychological resilience and reducing the probability of psychological avoidance (Liu et al., 2025a). On the other hand, it may enhance psychological belonging through emotional support, thus alleviating loneliness and life stress (Gür and Gür, 2025).

**Figure 1:**
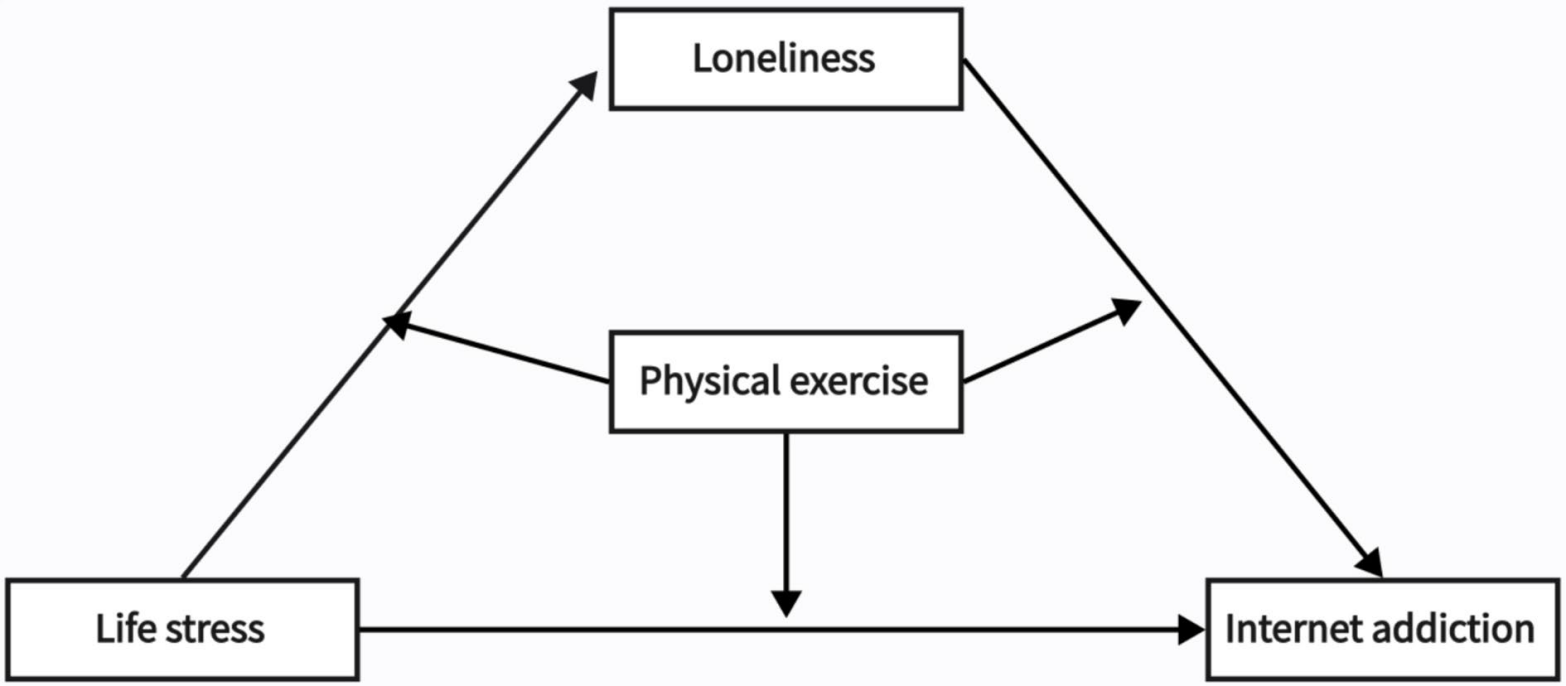
Theoretical hypothetical model diagram.

Based on the above research viewpoints, this study proposes Hypothesis 2: Physical exercise plays a moderating role in all paths of “college students’ life stress → Internet addiction” and “life stress → loneliness → Internet addiction”. Schematic diagram of the overall model of Hypothesis 1 and Hypothesis 2 (Figure 1).

## 3 Research methods

### 3.1 Participants and procedures

This study has been approved by the Theoretical Review Committee of Hubei University of Automotive Technology (2026LLSC11). Undergraduate students from Heze University (Shandong), Hanjiang Normal University (Hubei), and Kashi University (Xinjiang) were selected as survey subjects from the eastern, central, and western regions of China, respectively. Physical education teachers adopted the convenient sampling method and distributed questionnaires through the “WENJUANXING” online platform. Before distributing the questionnaires, participants were informed of the survey purpose, precautions, privacy protection measures, etc. All participants were asked to sign the informed consent form and were informed of their right to withdraw at any time. Based on the number of questions, it takes about 3–5 min to complete the questionnaire. Therefore, responses with too short completion time, consecutive identical answers, or incomplete responses were excluded. A total of 409 valid questionnaires were received, with an effective rate of 90.8%, including 183 male students and 226 female students (see Table 1).

**Table 1:** Demographic analysis.

Variety	Gender		Grade			
From	Male student	Female student	First-year university student	Second-year university student	Third-year university student	Fourth-year university student
Quantities	183	226	121	114	96	78
Percentage	44.7%	55.3%	29.6%	27.9%	23.5%	19.0%
PE (*M* ± SD)	17.050 ± 17.019	11.320 ± 13.061	14.770 ± 15.705	8.460 ± 11.911	18.570 ± 16.256	14.690 ± 15.321
LS (*M* ± SD)	3.052 ± 0.546	3.048 ± 0.556	2.952 ± 0.524	3.131 ± 0.517	3.048 ± 0.563	3.092 ± 0.608
LL (*M* ± SD)	2.872 ± 0.630	3.013 ± 0.662	2.832 ± 0.596	3.084 ± 0.660	2.921 ± 0.640	2.972 ± 0.704
IA (*M* ± SD)	2.962 ± 0.625	3.031 ± 0.617	2.907 ± 0.541	3.108 ± 0.673	2.983 ± 0.638	3.009 ± 0.623

### 3.2 Measurement tools

#### 3.2.1 Perceived Stress Scale

The Chinese version (CPSS) of the Perceived Stress Scale (PSS) developed by Cohen et al. (1983) and later translated and modified by Yang and Huang (2003) was used. This scale consists of 14 items, divided into a sense of tension (e.g., “In the past month, I felt uneasy about unexpected things”) and a sense of loss of control (e.g., “In the past month, I felt that things didn’t go as I wished”). The scale uses a 5-point Likert scale, with 7 reverse-scored items. The effect value of the variable is taken from the average score of the items. A higher score indicates greater personal stress. Previous studies have shown that the Crobach’s *α* coefficient of this scale is 0.917, KMO = 0.839, Bartlett *p* < 0.001, and the test-retest reliability (ICC) is 0.78 (*p* < 0.001). Therefore, it indicates that the reliability and validity of this questionnaire are good, meeting the requirements of scientific research (Gavurova et al., 2022).

#### 3.2.2 Internet Addiction Scale

The Revised Chinese Internet Addiction Scale (CIAS-R) was developed by Bai and Fan (2005). The scale consists of 19 items, which are divided into core symptoms of Internet addiction (e.g., My life would be meaningless without the Internet) and Internet-addiction-related problems (e.g., I have slept less than 4 h for multiple times due to Internet use). Internet addiction symptoms include withdrawal reactions and tolerance, and Internet-addiction-related problems include interpersonal and health problems as well as time management problems (Yang et al., 2022b). It is a 5-point Likert scale, and the average score of each item represents the effect value of Internet addiction. Previous studies have shown that the Crobach’s *α* coefficients of the four factors within the scale are 0.81, 0.73, 0.79, and 0.73 respectively, and the Crobach’s *α* of the total scale’s internal consistency is 0.90. In the validity test, the correlation coefficients with Young’s Internet Addiction Diagnostic Questionnaire and Morahan–Martin’s Pathological Internet Use Scale are 0.81 and 0.77, respectively. Therefore, it indicates that the questionnaire has good reliability and validity (Monteiro et al., 2023).

#### 3.2.3 Loneliness Scale

In this study, the Chinese abbreviated version of the UCLA Loneliness Scale (ULS-6) (Wang and Zeng, 2024b; Chen et al., 2025) was selected. This questionnaire consists of a total of 6 items. For example, “Do you often feel that you lack a partner?” and “Do you often feel ignored?” The scale is composed of a 5-point Likert scale. The loneliness score is calculated as the average value of each item. The higher the score, the stronger the level of loneliness. Previous studies have shown that for this questionnaire, Crobach’s *α* = 0.942, the CR reliability is 0.861, and the KMO value is 0.768, indicating that the questionnaire has good reliability and validity (Ge et al., 2025).

#### 3.2.4 Physical Exercise Scale

The classic Physical Activity Rating Scale (PARS - 3), revised by Deqing (1994), was adopted. This scale consists of three items, evaluating the intensity, frequency, and duration of physical activity. Its calculation formula is Intensity * (Time − 1) * Frequency, and the total score ranges from 0 to 100 points (Guan et al., 2025). Based on the standard that moderate physical exercise can effectively alleviate stress, the physical exercise levels are divided into three groups: low (≤19), moderate (20–42), and high (≥43). This scale has been widely used in the investigation of the physical exercise volume of Chinese college students and is indicated to have good reliability and validity (Jiang and Bian, 2025). Research shows that Crobach’s *α* = 0.712, the KMO value is 0.719, the Bartlett test *p* < 0.001, and CR = 0.836. Although Crobach’s *α* is lower than that of other scales in this study, it is because this scale only has three items and measures only behavioral facts, and its reliability level is still within the acceptable range (Lee and Comrey, 1978). Moreover, previous studies have confirmed that this questionnaire has good structural validity (Ding et al., 2021).

### 3.3 Data analysis

In this study, SPSS 28.0 software was used for data collection and common method bias test. Descriptive statistics and correlation tests were conducted on the data. Based on the results of the correlation test, pytharm 2025.2.0.1 was used to generate a correlation heatmap. The mediation effect of loneliness (Model 4) and the moderating effect of physical exercise (Model 59) were analyzed through the PROCESS 4.1 plug-in. Then, with the help of functions such as syntax editing, a moderated model diagram was generated.

## 4 Results

### 4.1 Bias testing of commonly used methods

To rule out the issue of common method bias, the Harman single-factor test was conducted on the measurement data. An un-rotated principal component analysis of life stress, loneliness, Internet addiction, and physical exercise was performed using SPSS 28.0. The results showed that there were a total of 15 factors with eigenvalues greater than 1. The explanatory rate of the first factor was 36.6%, which was less than the critical value of 40%, indicating that there was no common method bias in the data of this study.

### 4.2 Descriptive statistics and correlation analysis

Descriptive analysis statistics show (Table 1) that the sample consists of a total of 409 college students, with males and females accounting for 44.7 and 55.3% respectively, indicating that the number of male and female college students participating in the survey is approximately equal. Analyzing the proportion of students in each grade, there are 78 fourth-year students, accounting for 19.0%, which is the smallest proportion. However, the overall distribution is relatively uniform, meeting the requirements for the sample size distribution in scientific research. It is worth noting that there are significant differences in physical exercise among gender subgroups, with males having a higher amount of physical exercise than females (*p* < 0.001); there are also significant differences in physical exercise among grade subgroups, with the third-year students having the highest amount of physical exercise (*p* < 0.01). The reasons may be related to the social and cultural expectations of males, the learning tasks of different grades, and the cognition of physical exercise.

**Figure 2:**
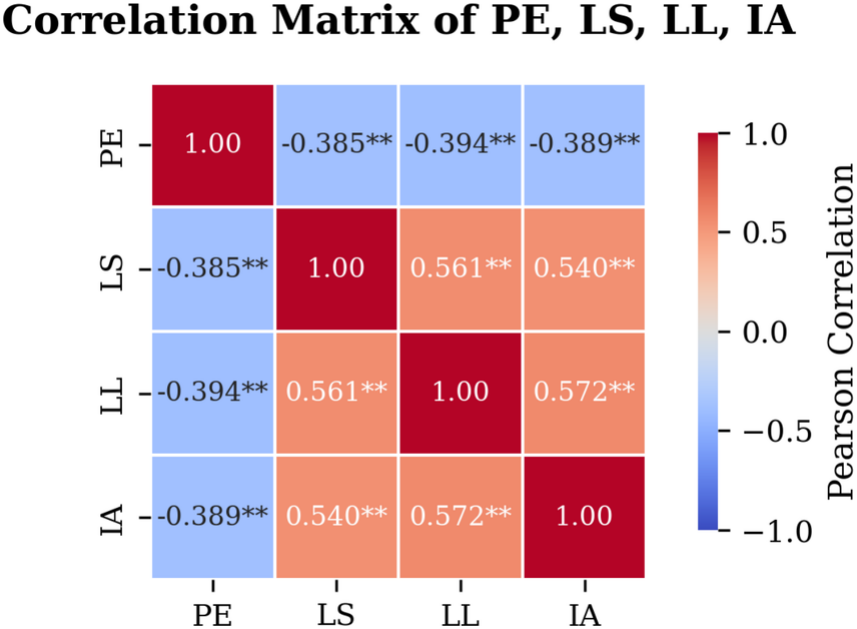
Pearson correlation heatmap among variables.

The results showed that there was a significant positive correlation between college students’ life stress and Internet addiction (*r* = 0.540, *p* < 0.01); there was a significant positive correlation between life stress and loneliness (*r* = 0.561, *p* < 0.01); there were significant negative correlations between physical exercise and life stress, Internet addiction, and loneliness (*r* = −0.385, *p* < 0.01; *r* = −0.389, *p* < 0.01; *r* = −0.394, *p* < 0.01). The correlation test indicated that there was a moderate association among the variables, which provided support for the scientific analysis of mediation and moderation (see Figure 2).

### 4.3 Mediating role of loneliness

**Figure 3:**
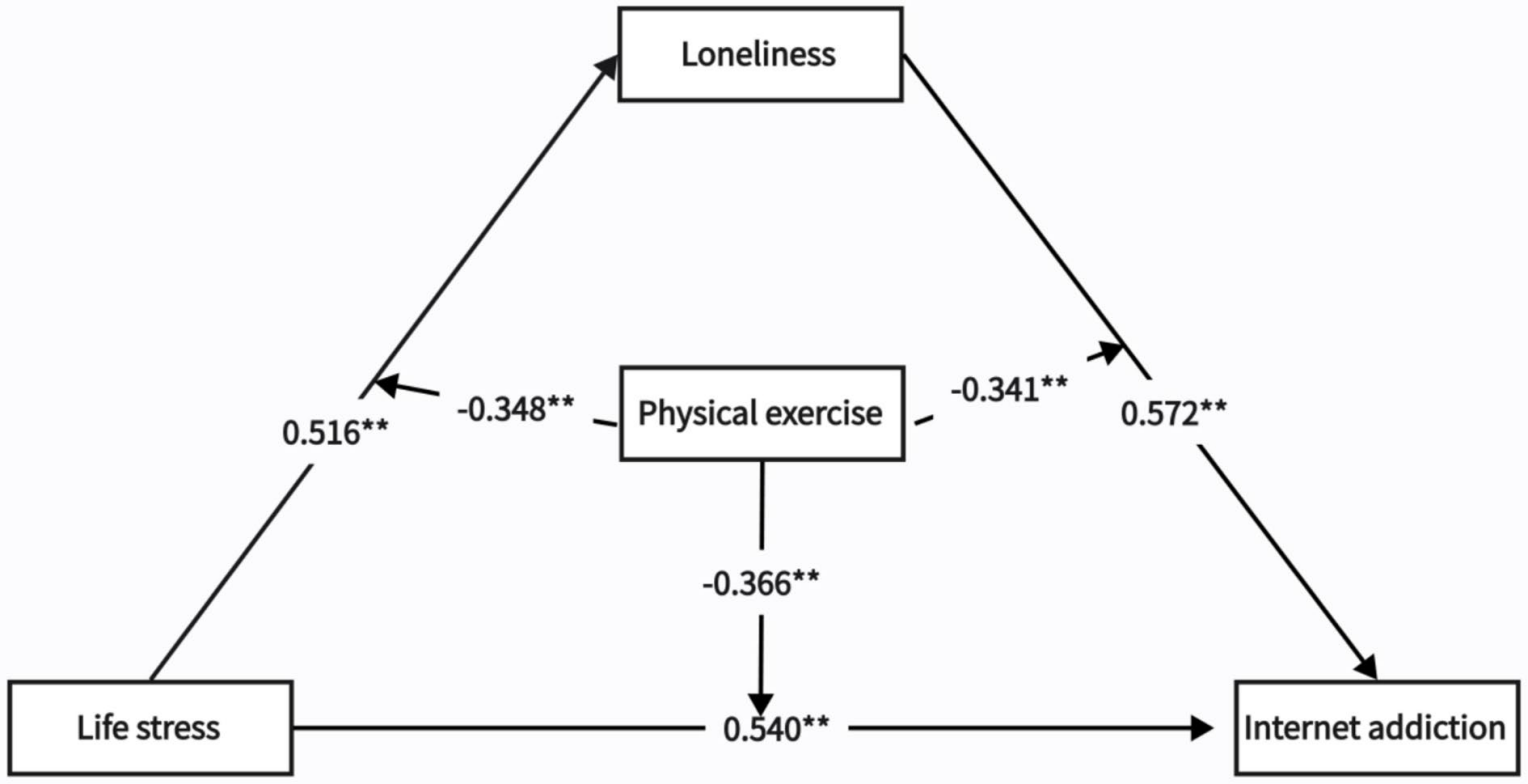
Moderated mediation model diagram of the influence of life stress on Internet addiction among college students; **p < 0.01.

A mediating effect test was conducted on the loneliness between life stress and Internet addiction, with gender and grade as control variables. Regression analysis showed that there was a significant positive correlation between life stress and loneliness (*β* = 0.516, *p* < 0.01), and a significant positive correlation between loneliness and Internet addiction (*β* = 0.572, *p* < 0.01) (Figure 3). Through 5,000 repeated sampling tests using the Bootstrap method, the mediating effect of loneliness between life stress and Internet addiction was shown (Table 2). The effect value of the mediating path was 0.248, accounting for 40.7% of the total effect, and the 95% confidence interval did not contain 0 (LLCI = 0.188, ULCI = 0.319). Therefore, loneliness plays a partial mediating role between life stress and Internet addiction.

**Table 2:** Analysis of mediation paths.

Path	Effect value	Proportion	SE	*t*	Bootstrap 95% CI	
					LLCI	ULCI
LS → IA (total)	0.609	100%	0.048	12.688	0.514	0.704
LS → LL → IA	0.248	40.7%	0.033	8.03	0.188	0.319
LS → IA (direct)	0.361	59.3%	0.053	6.491	0.256	0.465

### 4.4 Moderating effect of physical exercise

**Figure 4:**
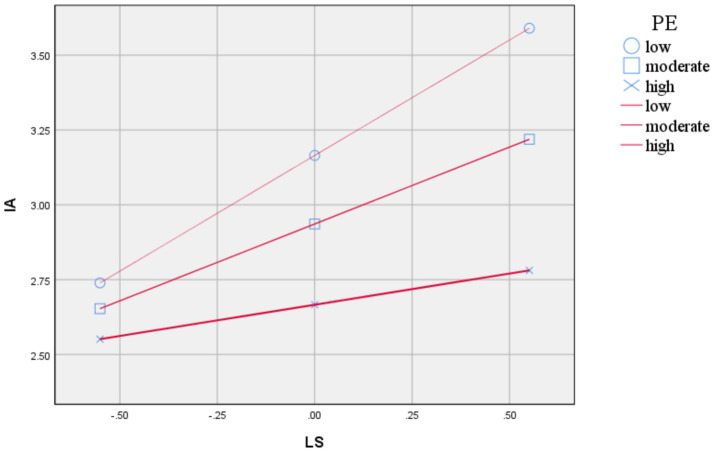
The relationship between life stress and internet addiction among college students with different levels of physical exercise.

Based on the hypothesized model, the moderating effects of physical exercise on three paths were tested after including gender and grade as control variables. The research results show that the interaction between life stress and physical exercise has a significant negative predictive effect on college students’ Internet addiction (*β* = −0.366, *p* < 0.01) (Figure 3), indicating that physical exercise negatively moderates the relationship between life stress and Internet addiction. In the Bootstrap test, the effect size of the interaction between life stress and physical exercise is −0.020 [95% CI (−0.018, −0.007)] (Table 3), further supporting the significant moderating effect of physical exercise. Specifically, when physical exercise volume is divided into three levels: low, medium, and high (scores: low ≤19, medium (20–42), high >43), it can be seen that for low-level physical exercise, the effect size of life stress on Internet addiction is 0.722 [95% CI (0.661, 0.883)], while for high-level physical exercise, the effect size is 0.209 [95% CI (0.109, 0.328)] (Table 4). From the relationship diagram between life stress and Internet addiction (Figure 4), the influence slopes of the two under different physical exercise volumes of low, medium, and high decrease in turn, indicating that the impact of life stress on Internet addiction is the smallest under high exercise volume. Therefore, it can be judged that physical exercise can weaken the impact of life stress on Internet addiction.

**Table 3:** Bootstrap test analysis of the relationships among model variables.

Result variable	Predictive variable	*R*	Effect value (*B*)	SE	*t*	Bootstrap 95% CI	
						LLCI	ULCI
Loneliness	Life stress	0.633	0.566	0.049	11.491	0.469	0.663
	Physical exercise		−0.016	0.002	−7.369	−0.020	−0.012
	LS*PE		−0.015	0.003	−5.705	−0.021	−0.010
Internet addiction	Loneliness	0.691	0.248	0.045	5.552	0.160	0.335
	Life stress		0.344	0.051	6.749	0.244	0.445
	LL*PE		−0.017	0.003	−3.179	−0.017	−0.004
	Physical exercise		−0.016	0.002	−7.316	−0.021	−0.012
	LS*PE		−0.020	0.003	−4.710	−0.018	−0.007

**Table 4:** The relationship between life stress and internet addiction among college students with different levels of physical exercise.

Group	Effect value	SE	*t*	Bootstrap 95% CI	
				LLCI	ULCI
Low level of PE	0.772	0.056	13.689	0.661	0.883
Moderate level of PE	0.514	0.046	11.112	0.423	0.605
High level of PE	0.209	0.060	3.458	0.109	0.328

In addition, the regression results of control variables show that the predictive effect of gender on loneliness is not significant (*β* = 0.078, *p* > 0.05), and the predictive effect of grade on loneliness is also not significant (*β* = 0.056, *p* > 0.05). The predictive effect of gender on Internet addiction is not significant (*β* = 0.039, *p* > 0.05), and the predictive effect of grade on Internet addiction is not significant either (*β* = 0.052, *p* > 0.05). This indicates that after controlling for gender and grade, the results of the main effect and interaction effect are stable.

**Figure 5:**
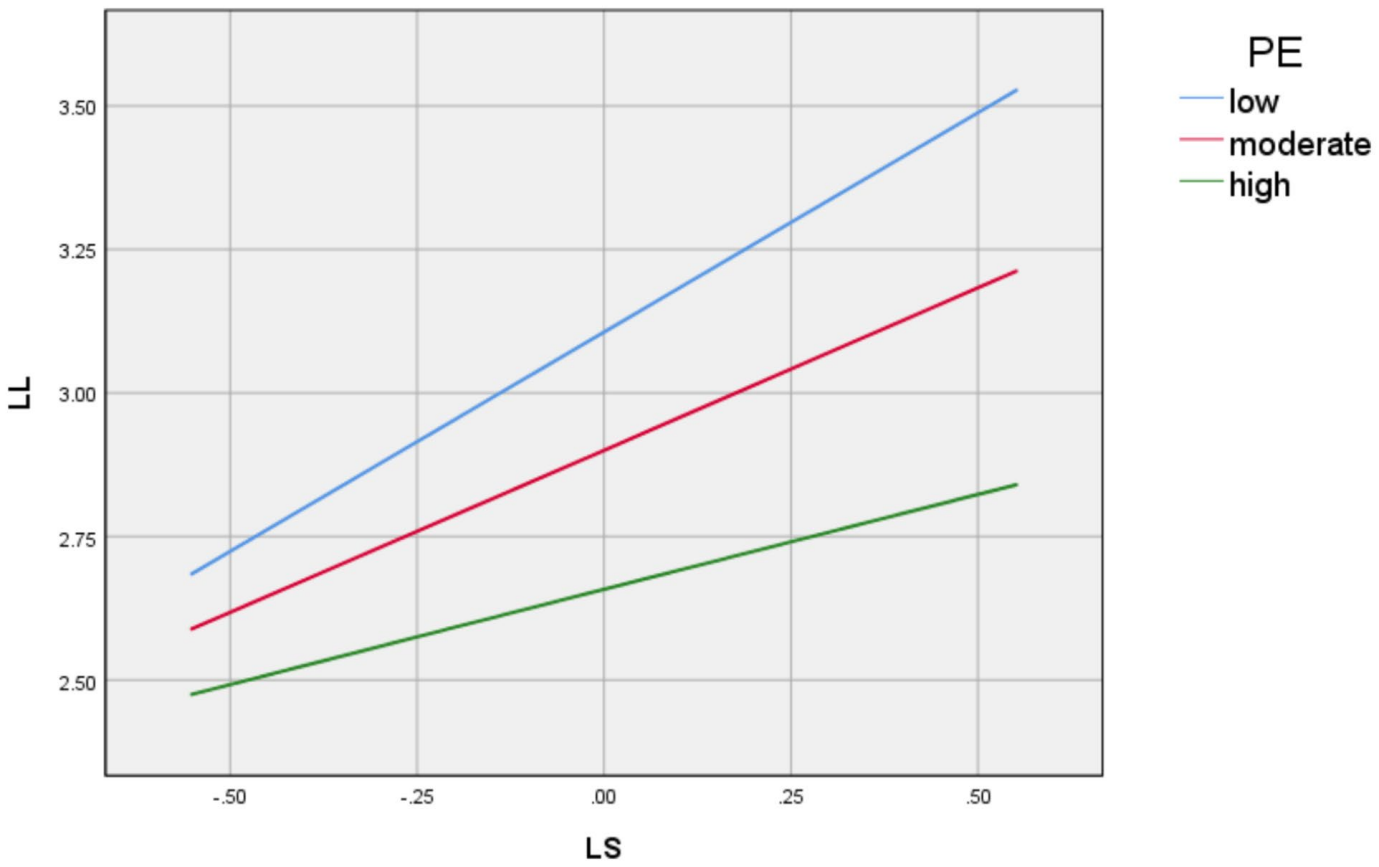
The relationship between life stress and loneliness among college students with different levels of physical exercise.

In addition, the interaction between life stress and physical exercise significantly predicted loneliness (*β* = −0.348, *p* < 0.01) (Figure 3). The effect size between them was −0.015 [95% CI (−0.021, −0.010)] (Table 3), indicating that physical exercise negatively moderated the relationship between college students’ life stress and loneliness. Under the low exercise level, the effect of life stress on loneliness was 0.765 [95% CI (0.647, 0.883)], and under the high exercise level, the effect of life stress on loneliness was 0.332 [95% CI (0.205, 0.458)] (Table 5). As can be seen from the relationship diagram between life stress and loneliness (Figure 5), the slopes of the effect between the two decreased successively under low, medium, and high physical exercise volumes, indicating that under the high physical exercise volume, the effect of life stress on loneliness was the smallest. Therefore, physical exercise can buffer the effect of college students’ life stress on loneliness.

**Table 5:** Relationship between life stress and loneliness among college students with different levels of physical exercise.

Group	Effect value	SE	*t*	Bootstrap 95% CI	
				LLCI	ULCI
Low level of PE	0.765	0.060	12.726	0.647	0.883
Moderate level of PE	0.566	0.049	11.491	0.469	0.663
High level of PE	0.332	0.064	5.151	0.205	0.458

**Figure 6:**
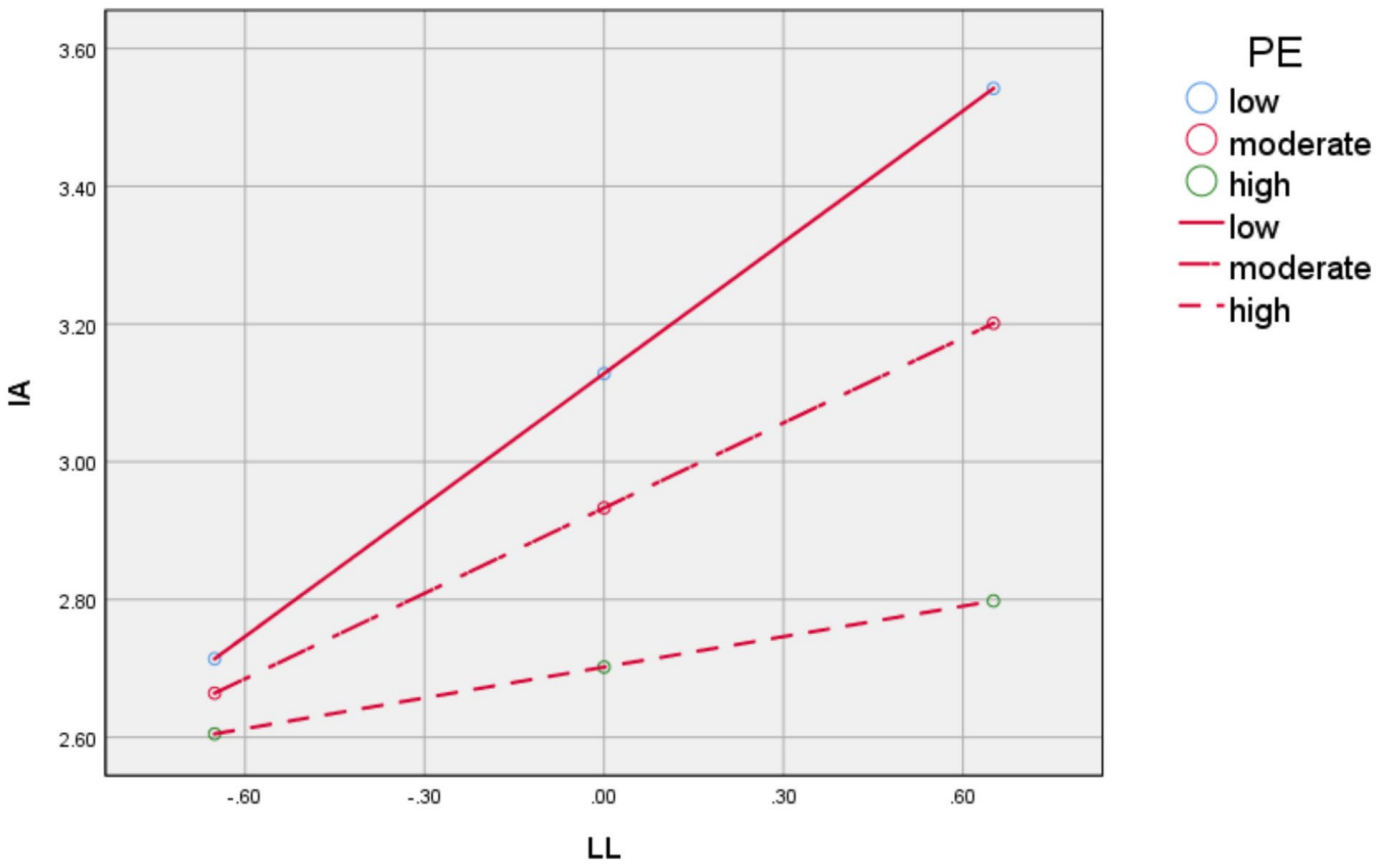
The relationship between loneliness and internet addiction among college students with different levels of physical exercise.

In addition, the interaction between loneliness and the level of physical exercise significantly predicted Internet addiction (*β* = −0.341, *p* < 0.01) (Figure 3). The effect size between the two was −0.017 [95% CI (−0.017, −0.004)] (Table 3), indicating that physical exercise negatively moderated the relationship between college students’ loneliness and Internet addiction. At a low exercise level, the effect size of loneliness on college students’ Internet addiction was 0.636 [95% CI (0.538, 0.734)], while at a high exercise level, the effect size of loneliness on college students’ Internet addiction was 0.148 [95% CI (0.007, 0.290)] (Table 6). As can be seen from the relationship diagram of loneliness and Internet addiction (Figure 6), the slopes of the two decreased successively under low, medium, and high exercise volumes, indicating that the influence of loneliness on Internet addiction was the smallest under a high exercise volume. Therefore, the role of physical exercise mitigated the influence of loneliness on Internet addiction. Overall, in the influence of college students’ life stress on Internet addiction, physical exercise played a moderating role in both the mediating path and the direct path, and physical exercise effectively alleviated the influence of life stress on Internet addiction (Figures 4–6).

**Table 6:** The relationship between loneliness and internet addiction among college students with different levels of physical exercise.

Group	Effect value	SE	*t*	Bootstrap 95% CI	
				LLCI	ULCI
Low level of PE	0.636	0.050	12.733	0.538	0.734
Moderate level of PE	0.412	0.041	9.957	0.331	0.494
High level of PE	0.148	0.072	2.064	0.007	0.290

## 5 Discussion

The research results indicate that loneliness plays a partial mediating role between college students’ life stress and Internet addiction, and physical exercise plays a moderating role in this mediating process (Figure 4–6). Different levels of physical exercise have different moderating effects. Specifically, at a high level of physical exercise, the influence of life stress on college students’ Internet addiction will weaken, which provides empirical support for physical exercise to effectively relieve life stress, reduce loneliness, and buffer Internet addiction.

### 5.1 The mediating role of loneliness in the influence of college students’ life stress on internet addiction

This study reveals a significant positive correlation between life stress and Internet addiction (*β* = 0.540, *p* < 0.01), which is consistent with the results of previous studies (Yan et al., 2022; Wang et al., 2024; Li et al., 2026). Life stress induces low mood, interpersonal conflicts, and intensified negative emotions through negative life events, thereby triggering Internet addiction (Gao et al., 2020; Mehmood et al., 2024). The stress and coping theory indicates that when facing life stress, college students are prone to generate more thinking burdens and fall into a state of anxiety, depression, and repeated self-depletion. To cope with this stress response, individuals often buffer psychological stress by avoiding reality and entering the virtual world (Codina et al., 2025). In real life, the most common way for college students to avoid stress and enter the virtual world is online games, which are easily accessible and low-cost, and can prompt the timely secretion of dopamine, bringing short-term pleasure (Shi and Wang, 2023). The study by Huang et al. (2025) points out that when facing academic pressure, employment pressure, and interpersonal conflicts, college students often choose online games or social media to relieve emotional distress. As time passes, it will intensify their dependence on the Internet to fill the inner void, ultimately leading to the development of Internet addiction.

Loneliness plays a partial mediating role in the path between college students’ life stress and Internet addiction. The mediating effect value is 0.248, accounting for 40.7%, which is consistent with previous research results (Wang and Gong, 2025; Zhou et al., 2025). As a subjective experience of unmet social needs, loneliness transforms external and emotional life stress into internal signals of emotional deficiency (Nibal, 2023), driving individuals to relieve inner discomfort by overusing the Internet. It is the main path of stress-response (Ge et al., 2025). This study found that the direct path (0.361) between life stress and Internet addiction is greater than the indirect path (0.248), indicating that life stress mainly leads to Internet addiction through the direct path. This conclusion is consistent with previous research, that is, stress can directly trigger the overuse of Internet devices and services, and its driving force comes from the immediate escape and coping mechanism (Chen et al., 2025). In the actual survey, it was found that the overuse of Douyin short-videos and online games is relatively high, which greatly affects the daily routine and learning quality of college students, leading to procrastination. This vicious behavioral cycle further exacerbates loneliness. Therefore, for practical intervention measures, it is necessary to dredge stress-venting channels, establish real-world social connections, and open up self-regulation paths to block the generation of loneliness, thereby reducing the risk of Internet addiction.

### 5.2 The moderating effect of physical exercise

The results of this study show that physical exercise has significant moderating effects on the relationships of “life stress → Internet addiction”, “life stress → loneliness”, and “loneliness → Internet addiction”, indicating that the protective effect of physical exercise is mainly manifested in the stages of stress response and psychological resource regulation.

This study further found that physical exercise plays a significant moderating role between college students’ life stress and Internet addiction. Under the condition of high physical exercise, the effect value of life stress on Internet addiction drops to 0.209, while under the condition of low physical exercise, this effect value is as high as 0.772. This indicates that physical exercise can effectively buffer the impact of college students’ life stress on Internet addiction. Based on the stress-coping theory, physical exercise is a positive behavioral coping strategy that can enhance individuals’ self-efficacy and social adaptability. College students who regularly participate in physical exercise have higher levels of self-efficacy and are more inclined to adopt active coping methods focusing on problem-solving rather than getting caught in complaining-style mental exhaustion (Orsolini et al., 2022; Lv et al., 2025b). From the perspective of executive function, regular physical exercise can improve the function of the prefrontal cortex, enhance the ability of impulse inhibition and the tendency of delayed gratification, thereby reducing the immediate gratification brought by Internet addiction (Lv et al., 2025a). Of course, it is also possible that physical exercise reduces Internet use by occupying time. However, overall, physical exercise is an effective way to regulate life stress and Internet addiction (Wang et al., 2024; Shiferaw et al., 2025).

This study also found that physical exercise plays a significant moderating role between college students’ life stress and loneliness. Under high-exercise levels, the effect value of life stress on loneliness drops to 0.332, while under low-exercise conditions, the effect value is 0.765. This indicates that physical exercise can effectively buffer the impact of life stress on loneliness, and this result is consistent with previous studies (Nibal, 2023; Gür and Gür, 2025). From the perspective of the conservation of resources theory, physical exercise, as a supplementary psychological resource, fills the inner void and loneliness through cooperation and emotional communication within the team (Greco et al., 2024). Additionally, reasonable physical exercise is an effective way to relieve life stress (Chen et al., 2025), and team sports are important channels for expressing emotions, increasing interpersonal communication, and reducing loneliness (Zhu et al., 2025). Nowadays, college students are faced with employment pressure, academic pressure, and interpersonal relationship pressure, which are important sources leading to unstable personal emotions and even depression and rebellious psychology. Reasonable physical exercise is not only an important way to enhance human body functions but also a direct driving force for giving people confidence and self-efficacy when facing difficulties (Wang et al., 2025).

Finally, this study confirmed that physical exercise significantly moderated the relationship between college students’ loneliness and Internet addiction. Under the condition of high-level physical exercise, the effect value of loneliness on Internet addiction decreased to 0.148, while under the low-level physical exercise, the effect value was 0.636. This result indicates that regular physical exercise can effectively weaken the influence of college students’ loneliness on Internet addiction, presenting a “mitigating moderation” model. From the analysis of psychological mechanisms, the emotion regulation theory emphasizes that physical exercise is an effective non-pharmacological means to improve negative emotions (Li et al., 2026). Loneliness is essentially an aversive emotional experience, and individuals often seek immediate emotional relief by surfing the Internet. Regular physical exercise can effectively increase the levels of endogenous opioid peptides and dopamine, produce a positive emotion substitution effect, and reduce the motivation of individuals to “relieve worries through the Internet” (Greco et al., 2024). In addition, from the perspective of social connection, the core of loneliness lies in the lack of a sense of belonging (Heidari et al., 2025). Physical exercise provides a practical field for social interaction and emotional catharsis, enabling college students to establish peer support and emotional connections, compensating for the lack of personal sense of belonging. The realistic emotional substitution cuts off the chain of excessive Internet use.

Notably, the effect value (0.636) of loneliness on Internet addiction among individuals with low exercise volume is much higher than that (0.148) among those with high exercise volume. This population has become a high-risk group for Internet addiction. Currently, there is a widespread vicious cycle of “sedentary behavior → loneliness → Internet use → more loneliness” among Chinese college students, and physical exercise is precisely the effective fulcrum to break this cycle (Dong et al., 2024). Mental health interventions in higher education institutions should focus on promoting “integration of physical exercise into daily life” and regard exercise prescriptions as a high-efficiency strategy to block loneliness and Internet addiction and relieve life stress.

## 6 Conclusion

This study established a moderated mediation model and demonstrated that loneliness plays a partial mediating role between college students’ life stress and Internet addiction. Physical exercise plays a moderating role in this model, moderating the relationships among the paths of life stress and Internet addiction, life stress and loneliness, and loneliness and Internet addiction. It reveals that college students’ physical exercise can effectively alleviate the influence of life stress and loneliness on Internet addiction, providing a theoretical basis and practical strategies for current mental health intervention and Internet addiction prevention among college students.

### 6.1 Limitations and future prospects

Although this study has made some important discoveries, there are still some limitations. First, being a cross-sectional study, it lacks the time dimension, which restricts causal inference between dimensions and the capture of dynamic data changes. The static correlations between dimensions cannot completely rule out the influence of individual characteristics. In future research, more attention should be paid to longitudinal tracking and experimental design to make causal prediction more scientific and reasonable. Second, although the survey subjects are from universities in different regions of eastern, central, and western China, they are still influenced by China’s unique culture, values, and family expectations. For example, strong individual vanity, excessive family expectations, and strict academic requirements may lead to significant differences between Chinese college students and their Western counterparts in terms of life stress, loneliness, and Internet addiction. Therefore, the research conclusions have certain reference value, but their direct generalization is limited. In subsequent research, cross-cultural samples need to be included to verify the cultural universality of this model. Finally, there are numerous factors influencing Internet addiction. Variables such as the major of study, economic level, and sleep quality should be further incorporated as control variables, and stepwise regression analysis should be used to further explore the relationship between independent and dependent variables.

## Data Availability

The original contributions presented in the study are included in the article/supplementary material, further inquiries can be directed to the corresponding author.

## References

[ref1] Bai Y. Fan F. M. (2005). A study on the internet dependence of college students: the revising and applying of a measurement. Psychol. Dev. Educ. 4, 99–104. Available online at: https://devpsy.bnu.edu.cn/CN/Y2005/V21/I4/99

[ref2] Bakioglu F. (2020). Internet addiction and social self-efficacy: the mediator role of loneliness. Anal. Psicol. 36, 435–442. doi: 10.6018/analesps.394031

[ref3] Chen M. Y. Li-Ya A. Jiang Y. Y. Huang H. T. Liu S. Ma Y. . (2025). Dose-response relationship of loneliness and internet addiction with depression among college students: a multicenter survey. Int. J. Soc. Psychiatry 72, 571–581. doi: 10.1177/00207640251371433, 41159437

[ref4] Chen M. Zhang X. (2024). Factors influencing internet addiction among university students: the mediating roles of self-control and anxiety. Acta Psychol. 250:104535. doi: 10.1016/j.actpsy.2024.104535, 39442385

[ref5] Codina N. Pestana J. V. Ogden R. Wittmann M. Martin-Soelch C. Witowska J. . (2025). Problematic internet use predicts lesser satisfaction with life, but psychological distress acts as a mediator. Cyberpsychol. Behav. Soc. Netw. 28, 616–622. doi: 10.1177/21522715251365537, 40796321

[ref6] Cohen S. Kamarck T. Mermelstein R. (1983). A global measure of perceived stress. J. Health Soc. Behav. 24, 385–396. doi: 10.2307/2136404 6668417

[ref7] Deqing L. (1994). The stress levels of college students and their relationship with physical exercise. Chin. J. Mental Health 1, 5–6. Available online at: https://xueshu.baidu.com/ndscholar/browse/detail?paperid=d8903ab65f69f5d550d497b1e8f13ea8&site=xueshu_se

[ref8] Ding Z. C. Yan J. Fu J. (2021). Internet and mobile phone addiction self-control mediate physical exercise and subjective well-being in young adults using IoT. Mob. Inf. Syst. 2021:9923833. doi: 10.1155/2021/9923833

[ref9] Dong W. L. Tang H. S. Wu S. J. Lu G. L. Shang Y. Q. Chen C. R. (2024). The effect of social anxiety on teenagers’ internet addiction: the mediating role of loneliness and coping styles. BMC Psychiatry 24:395. doi: 10.1186/s12888-024-05854-5, 38802784 PMC11129444

[ref10] Gao L. L. Gan Y. Q. Whittal A. Lippke S. (2020). Problematic internet use and perceived quality of life: findings from a cross-sectional study investigating work-time and leisure-time internet use. Int. J. Environ. Res. Public Health 17:4056. doi: 10.3390/ijerph17114056, 32517203 PMC7311972

[ref11] Gavurova B. Ivankova V. Rigelsky M. Mudarri T. (2022). Internet addiction in socio-demographic, academic, and psychological profile of college students during the COVID-19 pandemic in the Czech Republic and Slovakia. Front. Public Health 10:944085. doi: 10.3389/fpubh.2022.944085, 35812472 PMC9260220

[ref12] Ge M. W. Hu F. H. Jia Y. J. Tang W. Zhang W. Q. Zhao D. Y. . (2025). The relationship between loneliness and internet or smartphone addiction among adolescents: a systematic review and meta-analysis. Psychol. Rep. 128, 1429–1451. doi: 10.1177/00332941231180119, 37261719

[ref13] Greco F. Quinzi F. Papaianni M. C. Cosco L. F. Segura-Garcia C. Emerenziani G. P. (2024). Effects of school-based physical activity on volition in exercise, sleep quality and internet addiction in Italian adolescents. Heliyon 10:e32129. doi: 10.1016/j.heliyon.2024.e32129, 38882324 PMC11177120

[ref14] Guan T. Sheng J. Tan L. (2025). Physical exercise and subjective well-being of Chinese adults: relationship and mechanisms based on the Chinese general social survey 2021. Soc. Behav. Pers. 53:e13344. doi: 10.2224/sbp.13344

[ref15] Gür F. Gür G. C. (2025). The relationship between physical activity and problematic internet use in Turkish college students: the chain-mediated role of self-control and distress. Psychiatry Q. 96, 641–663. doi: 10.1007/s11126-025-10133-x, 40180754 PMC12460438

[ref16] Heidari Z. Kianimoghadam A. S. Masjedi Arani A. Bakhtiari M. (2025). Investigating the relationship between loneliness, physical activity, and internet addiction: the mediating role of academic burnout and self-control. Iran. J. Psychiatry 20, 330–344. doi: 10.18502/ijps.v20i3.19040, 41185675 PMC12579799

[ref17] Huang Q. Y. Li J. J. Wang J. Y. Liu B. (2025). Negative life events and internet addiction among college students: role of physical exercise and prosocial behavior. Front. Psychol. 16:1629818. doi: 10.3389/fpsyg.2025.1629818, 41040106 PMC12484130

[ref18] Jiang Y. Bian T. Q. (2025). The effects of physical exercise on college students’ pro-social behavior: the chain mediating role of sense of meaning in life and subjective well-being. Front. Psychol. 16:1604700. doi: 10.3389/fpsyg.2025.1604700, 40606900 PMC12213650

[ref19] Koktas N. C. Keskin G. Yigitoglu G. T. (2024). Evaluation of internet addiction and relational variables among nursing students in Turkey during the COVID-19 pandemic. Health Educ. Behav. 51, 388–399. doi: 10.1177/10901981241230497, 38345029

[ref20] Lee H. B. Comrey A. L. (1978). An empirical comparison of two minimum residual factor extraction methods. Multivariate Behav. Res. 13, 497–507. doi: 10.1207/s15327906mbr1304_9, 26810747

[ref21] Li R. Fu W. Q. Liang Y. Q. Huang S. H. Xu M. Y. Tu R. (2024). Exploring the relationship between resilience and internet addiction in Chinese college students: the mediating roles of life satisfaction and loneliness. Acta Psychol. 248:104405. doi: 10.1016/j.actpsy.2024.104405, 39067239

[ref22] Li R. Li H. Y. Zuo H. J. Sun J. M. Liu Q. Li C. X. . (2026). The impact of college students’ physical exercise on negative emotion: the chain mediating role of self-efficacy and smartphone addiction. PLoS One 21:e0338382. doi: 10.1371/journal.pone.0338382, 41642928 PMC12875471

[ref23] Liu Y. Tong Y. Huang G. H. (2025b). Physical exercise and internet addiction among college students: chain mediation of social anxiety and loneliness. Soc. Behav. Pers. 53:e14762. doi: 10.2224/sbp.14762

[ref24] Liu Y. Yin J. Xu L. Luo X. Liu H. Zhang T. (2025a). The chain mediating effect of anxiety and inhibitory control and the moderating effect of physical activity between bullying victimization and internet addiction in Chinese adolescents. J. Genet. Psychol. 186, 397–412. doi: 10.1080/00221325.2025.2462595, 39921534

[ref25] Luo M. Duan Z. Chen X. (2024). The role of physical activity in mitigating stress-induced internet addiction among Chinese college students. J. Affect. Disord. 366, 459–465. doi: 10.1016/j.jad.2024.08.188, 39216640

[ref26] Lv Y. Sheng L. Gao J. J. Chen M. S. Xin S. F. (2025b). The longitudinal relationship between stressful life events and adolescent internet addiction: a life history theory perspective. Curr. Psychol. 44, 17135–17147. doi: 10.1007/s12144-025-08375-w

[ref27] Lv Y. Yin Y. W. Liu Y. (2025a). Chain mediated effects of stress perception and loneliness on the relationship between physical exercise and internet addiction among chemistry majors in college. Sci. Rep. 15:45149. doi: 10.1038/s41598-025-33412-w, 41429938 PMC12749627

[ref28] Maes M. Vanhalst J. Spithoven A. W. Van Den Noortgate W. Goossens L. (2016). Loneliness and attitudes toward aloneness in adolescence: a person-centered approach. J. Youth Adolesc. 45, 547–567. doi: 10.1007/s10964-015-0354-5, 26369350

[ref29] Mehmood K. Suhail A. Kautish P. Hakeem M. M. Rashid M. (2024). Turning lemons into lemonade: social support as a moderator of the relationship between technostress and quality of life among university students. Psychol. Res. Behav. Manag. 17, 989–1006. doi: 10.2147/prbm.S448989, 38495088 PMC10941796

[ref30] Monteiro A. P. Sousa M. Correia E. (2023). Internet addiction: relationship with anxiety, depression, stress and time online. Rev. Psicol. 16, 45–61. doi: 10.21615/cesp.6255

[ref31] Mousoulidou M. Christodoulou A. Averkiou E. Pavlou I. (2024). Internet and social media addictions in the post-pandemic era: consequences for mental well-being and self-esteem. Soc. Sci. Basel 13:699. doi: 10.3390/socsci13120699, 30654563

[ref32] Netz Y. Raviv S. (2002). Exercise, fitness, and subjective measures related to fitness of physical education and other teachers. Percept. Mot. Skills 94, 1091–1100. doi: 10.2466/pms.2002.94.3c.1091, 12186230

[ref33] Nibal S. (2023). Loneliness as a predictor of internet addiction among adolescents from Arab society in Israel. Edmetic 12:2. doi: 10.21071/edmetic.v11i1.15872

[ref34] Orsolini L. Yilmaz-Karaman I. G. Longo G. Bellagamba S. Kato T. A. Volpe U. (2022). Sex-differences in hikikomori traits as predictors of problematic internet use in Italian university students. J. Psychiatr. Res. 155, 211–218. doi: 10.1016/j.jpsychires.2022.08.015, 36075117

[ref35] Pierce L. L. Wilkinson L. K. Anderson J. (2003). Analysis of the concept of aloneness. As applied to older women being treated for depression. J. Gerontol. Nurs. 29, 20–25. doi: 10.3928/0098-9134-20030701-06, 12874936

[ref36] Shen X. W. Wang C. G. Chen C. Y. Wang Y. F. Wang Z. N. Zheng Y. P. . (2023). Stress and internet addiction: mediated by anxiety and moderated by self-control. Psychol. Res. Behav. Manag. 16, 1975–1986. doi: 10.2147/prbm.S411412, 37284553 PMC10239643

[ref37] Shi X. X. Wang R. L. (2023). School victimization and internet addiction among Chinese adolescents: the mediating roles of life satisfaction and loneliness. Front. Psychol. 13:1059486. doi: 10.3389/fpsyg.2022.1059486, 36710833 PMC9878454

[ref38] Shiferaw B. D. Tang J. Wang Y. Wang Y. Wang Y. Mackay L. E. . (2025). Impact of digital addiction on youth health: a systematic review and meta-analysis. J. Behav. Addict. 14, 1129–1158. doi: 10.1556/2006.2025.00081, 40928886 PMC12486297

[ref39] Song I. Larose R. Eastin M. S. Lin C. A. (2004). Internet gratifications and internet addiction: on the uses and abuses of new media. Cyberpsychol. Behav. 7, 384–394. doi: 10.1089/cpb.2004.7.384, 15331025

[ref40] Wang X. Y. Ding T. Lai X. B. Yang C. W. Luo J. H. (2024). Negative life events, negative copying style, and internet addiction in middle school students: a large two-year follow-up study. Int. J. Ment. Heal. Addict., 22, 3233–3243. doi: 10.1007/s11469-023-01045-7, 37363770 PMC10047474

[ref41] Wang Y. T. Gong G. D. (2025). Exploring the dynamic interactions between internet addiction, anxiety, and loneliness in adolescents: a longitudinal cross-lagged panel model. Front. Psych. 16:1705792. doi: 10.3389/fpsyt.2025.1705792, 41446295 PMC12723513

[ref42] Wang J. Tang L. Liu Y. Wu X. Zhou Z. Zhu S. (2025). Physical activity moderates the mediating role of depression between experiential avoidance and internet addiction. Sci. Rep. 15:20704. doi: 10.1038/s41598-025-07487-4, 40596572 PMC12217329

[ref43] Wang Y. Zeng Y. (2024). Relationship between loneliness and internet addiction: a meta-analysis. BMC Public Health 24:858. doi: 10.1186/s12889-024-18366-4, 38504216 PMC10953128

[ref44] Xu J. S. Tang L. Q. (2024). The relationship between physical exercise and problematic internet use in college students: the chain-mediated role of self-control and loneliness. BMC Public Health 24:1719. doi: 10.1186/s12889-024-19226-x, 38937729 PMC11212378

[ref45] Yan E. Sun R. W. Wu A. M. S. Lai D. W. L. Lee V. W. P. (2022). The impact of pandemic-related life stress on internet gaming: social cynicism and gaming motivation as serial mediators. Int. J. Environ. Res. Public Health 19:8332. doi: 10.3390/ijerph19148332, 35886180 PMC9316489

[ref46] Yang T. Z. Huang H. (2003). An epidemiological study on the psychological stress of urban residents during social transformation. Chin. J. Epidemiol. 9, 11–15. Available online at: http://chinaepi.icdc.cn/zhlxbx/ch/reader/view_abstract.aspx?file_no=20030906&flag=1

[ref47] Yang X. M. Wu X. C. Qi J. J. Zhou X. (2022b). Posttraumatic stress symptoms, adversity belief, and internet addiction in adolescents who experienced a major earthquake. Curr. Psychol. 41, 3013–3020. doi: 10.1007/s12144-020-00816-y

[ref48] Yang Q. Wu Z. H. Yang X. Z. Jiang S. H. Wu D. Oliffe J. L. (2022a). Associations between uncertainty stress, life stress and internet addiction among medical students. Front. Public Health 9:809484. doi: 10.3389/fpubh.2021.809484, 35141191 PMC8818744

[ref49] Ye Z. Zhang T. Peng Y. Rao W. Jia P. (2025). Effects of physical activity on smartphone addiction in Chinese college students-chain mediation of self-control and stress perception. BMC Public Health 25:1532. doi: 10.1186/s12889-025-22720-5, 40275263 PMC12020245

[ref50] Zheng X. T. Xue C. Chen J. W. (2026). Latent profile analysis of cumulative stress and protective factors in junior high school students’ problematic internet use. Int. J. Ment. Heal. Addict. 24, 753–774. doi: 10.1007/s11469-024-01441-7

[ref51] Zhou X. N. Amran M. S. Surat S. Yin H. (2025). Loneliness and social withdrawal among college students: the mediating role of internet addiction and the moderating effect of sex. Adolescents 5:51. doi: 10.3390/adolescents5040051

[ref52] Zhu Y. Du Z. Zhang Y. Li X. Meng J. (2025). A study of the symptom network structure of internet addiction, non-suicidal self-injury and internalization problems in adolescents and its association with physical activity. BMC Public Health 25:4065. doi: 10.1186/s12889-025-25370-9, 41266999 PMC12632008

